# Population density and habitat use of two sympatric small cats in a central Indian reserve

**DOI:** 10.1371/journal.pone.0233569

**Published:** 2020-06-04

**Authors:** Nilanjan Chatterjee, Parag Nigam, Bilal Habib

**Affiliations:** Wildlife Institute of India, Dehradun, Uttarakhand, India; Sichuan University, CHINA

## Abstract

Despite appreciable advances in carnivore ecology, studies on small cats remain limited with carnivore research in India being skewed towards large cats. Small cats are more specialized than their larger cousins in terms of resource selection. Studies on small cat population and habitat preference are critical to evaluate their status to ensure better management and conservation. We estimated abundance of two widespread small cats, the jungle cat, and the rusty-spotted cat, and investigated their habitat associations based on camera trap captures from a central Indian tiger reserve. We predicted fine-scale habitat segregation between these sympatric species as a driver of coexistence. We used an extension of the spatial count model in a Bayesian framework approach to estimate the population density of jungle cat and rusty-spotted cat and used generalized linear models to explore their habitat associations. Densities of rusty-spotted cat and jungle cat were estimated as 6.67 (95% CI 4.07–10.74) and 4.01 (95% CI 2.65–6.12) individuals/100 km^2^ respectively. Forest cover and evapotranspiration were positively associated with rusty-spotted cat occurrence whereas both factors had a significant negative relation with jungle cat occurrence. The results directed habitat segregation between these small cats with affinities of rusty-spotted cat and jungle cat towards well-forested and open scrubland areas respectively. Our estimates highlight the widespread applicability of this model for density estimation of species with no individual identification. Moreover, the study outcomes can aid in targeted management decisions and serve as the baseline for species conservation as these models allow robust population estimation of elusive species along with predicting their habitat preferences.

## Introduction

Carnivores naturally occur at low densities owing to their apex position in the food web. Concurrently they continue to face rapid population decline caused by contracting range sizes and fragmentation of existing habitat [1]. Asia holds more than 60% of the global diversity of cats [2], harbouring 21 out of 36 species. India, a mega-biodiverse country, is home to 15 among them [3]. The geographical distribution range of most of these cats lies within protected areas with an average size of less than 400 km^2^, which contributes to only five percent (http://wiienvis.nic.in/Database/Protected_Area_854.aspx) of the total landmass of the country. Furthermore, these protected areas are located within high densities of human populations dependent on local resources and surrounded by land which is undergoing an upsurge in anthropogenic developmental activities. Given these escalating pressures on felid species in Asia [4–6], an in-depth understanding of their population status and habitat use would be fundamental for designing effective conservation and management policies. Additionally, as a number of the species do not have unique pelage patterns, capture-recapture techniques [7] cannot be applied for population estimation and owing to their elusive nature, direct encounters are also rare. We addressed these issues using the spatial presence-absence model [8] based on field-based sampling using automated camera-traps.

Among the small cats native to India, the jungle cat (*Felis chaus*) and rusty-spotted cat (*Prionailurus rubiginosus*) are the two, most widespread species. The former belongs to the house cat lineage, whereas the latter to the leopard cat lineage [9]. Rusty-spotted cat, the smallest cat in the world (average body weight 1.6 kg), is also reported to use the arboreal habitat alongside the terrestrial domain [3]. However, no such adaptation has been reported for the jungle cat (average body weight of 5 kg). Both species are known to be nocturnal and elusive [3, 10]. The jungle cat is categorized as “Least Concern” in IUCN red list [11] due to its large distribution range from south-eastern Asia to middle east and eastern Europe, while the rusty-spotted cat was recently downgraded to “Near Threatened” from “Vulnerable”, owing to new records from previously unknown locations [12].

Although taxonomy and phylogeography of these small cats have been studied [13, 14], fundamental ecological information on parameters like demography, habitat use, and activity pattern is majorly lacking. Previous studies have not attempted to estimate the population of any of these species, nevertheless, the population trend of both the species is presumed to be decreasing [11,12]. Most studies on the rusty-spotted cat are based on opportunistic sightings [15, 16]. A single study discussed the habitat use, seasonal abundance [17], and distribution pattern [18] of small carnivores from one protected area in the Western Ghats. The study [17] had limited inference owing to a small sample size (n = 11) but indicated a positive relationship of the abundance of the rusty-spotted cat with deciduous forest and that of jungle cat with dry thorn forest.

In this context, as both species have overlapping distribution ranges, we aimed at predicting fine-scale segregation in a habitat that enables the coexistence of these sympatric small cats. We hypothesized that habitat segregation at a fine-scale between these two species as a major driver for the cooccurrence. Also, the population estimation would fill the existing knowledge gap of these species using camera-trap captures as our study is the first attempt to estimate the population density of these individually unidentifiable small cats.

## Materials and methods

### Study sites

We conducted the study in Tadoba-Andhari Tiger Reserve (TATR) (20°04´-28°025´N, 79°13´-79°33´E) in the state of Maharashtra in Central India. The reserve spreads across an area of 1700 km^2^ in the Deccan plateau. The terrain of TATR is mostly undulating and hilly in the north and flat in the southern part [19]. The climate is characterized by a hot and long summer and a short and mild winter [19]. Annual average precipitation is around 1,200 mm, received mainly from south-western monsoons between June and September [20]. The vegetation of the reserve has been classified as Southern Tropical Dry Deciduous forest [21] dominated by bamboo (*Dendrocalamus strictus*) and teak (*Tectona grandis*). The habitat is majorly homogenous comprising of dense forest cover in 68%, open forest in 10% and human habitation in 21% of the total area of the reserve.

More than 20 mammal species have been recorded from the reserve through camera-trapping studies [22]. These include four species of felids (tiger, leopard, jungle cat and rusty-spotted cat), an ursid (sloth bear), two species of canids (dhole, jackal) and one species of mustelid (honey badger). Wild pig and chital are the major prey species followed by sambar, nilgai and gaur.

### Field methods

**Fig 1:**
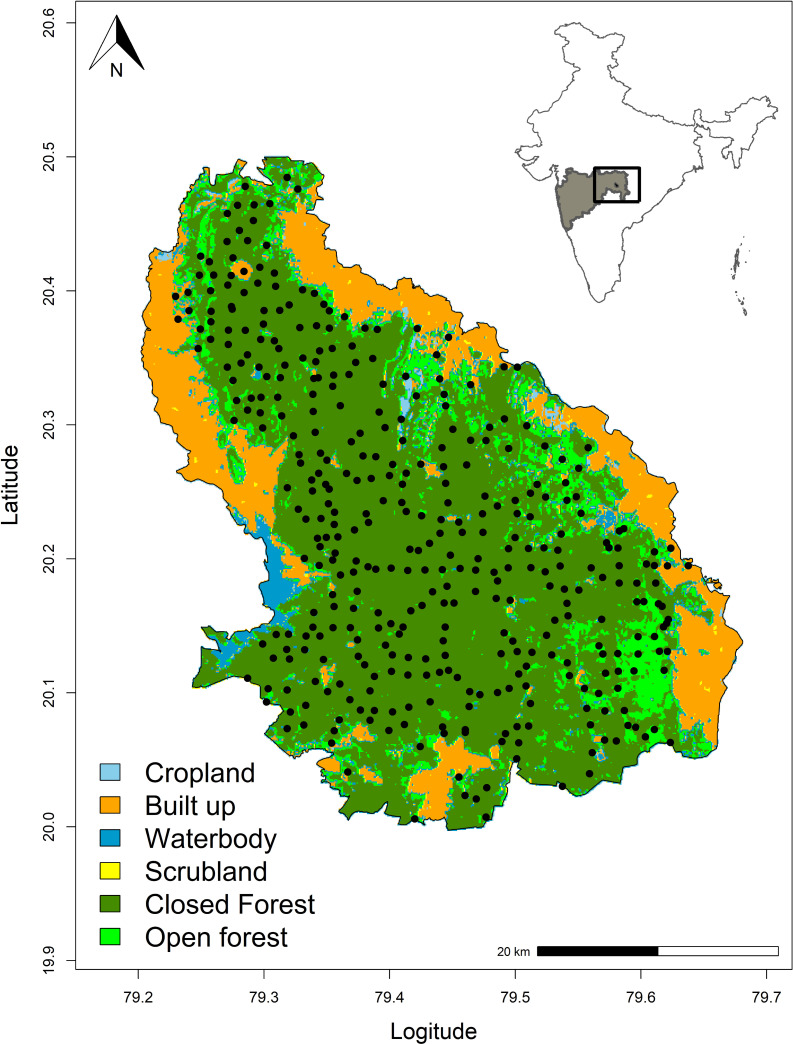
Study area showing camera trap locations in Tadoba-Andhari Tiger Reserve (TATR). Inset: Location of the study area in India. The map shows closed forest areas (in dark green), open forest (in light green), scrublands (in yellow), waterbodies (in blue) and human habitation (in orange) within the boundary of Tadoba-Andhari Tiger Reserve. Land-use data were obtained from Copernicus Global Land Service (https://land.copernicus.eu/) under CC 4.0 License.

We conducted camera-trap surveys in the dry season from February to June 2016. A pair of automated motion-triggered digital camera-traps (Cuddeback C1 or Cuddeback Ambush www.cuddeback.com) was deployed at 397 locations without using lure or bait (Fig 1). Due to the limited availability of camera-traps, sampling in the reserve was carried out in the tiger reserve dividing the whole area into four different blocks with an average of 100 stations per block. Cameras were shifted to consecutive blocks after sampling was done in one block. The cameras were deployed dividing the area following a grid size of 2 km^2^, equivalent to the home-range size (2–5 km^2^) of another small cat, the leopard cat which is widespread across the country [23]. Camera traps deployment was aimed for proportional representation of different habitat types (except human habitation) across the reserve. The average distance between two adjacent camera traps was 1.03 km, ensuring no large gaps in camera placement for the detection of individual small cats and to avoid pseudo-replication. Cameras were placed on both sides of roads, animal trails and fire-lines facing each other, placed around 30–40 cm above the ground. Camera-trap placement at trails optimizes the capture of large as well as small carnivores [24, 25]. Also, the placement of two camera-traps at every site increased the detectability of these small felids [26]. Cameras were programmed to take three photographs per trigger with an interval of 5 sec. Photographs taken within 30 min from the first trigger were not used for analysis to maintain the independence of captures. All the camera-traps were active for 24 hours continuously for 26–30 days and checked once in 15 days. Sampling interval was restricted to 26–30 days to ensure demographic closure [27].

### Density estimates

In this study individuals of neither species are uniquely identifiable from photographs. Therefore, we used spatial presence-absence (SPA) models [8], an extension of the spatial count model of [28]. Spatial count models the latent encounters of spatially referenced individuals with sampling devices using data augmentation and Markov chain Monte Carlo (MCMC) sampling in a Bayesian framework [8]. SPA models are structurally similar to spatial capture-recapture (SCR) models [7]. We assumed a half-normal detection function to model the probability of detection. Similar to SCR models, SPA models estimate g_0_ (baseline encounter rate), σ (scale/movement parameter related to home-range of the species) and N (population size) [8].

For each camera site, the detection or non-detection of these cats were recorded on each 24h sampling interval. The state-space (S) was comprised of the sampled area and a surrounding buffer area that is large enough to include all individuals potentially exposed to sampling. We estimated σ from the body-size and daily-movement distance equation [29]. We employed multiple buffer (σ, 2σ and 5σ) values around the state spaces to test the effect of buffer size on the density estimates of the SPA model. Following the equation of [29], the estimates of the home-range size of these cats were calculated as 0.8–1.2 km^2^ and 2–5 km^2^ for rusty-spotted cat and jungle cat respectively. A vague uniform prior, U (-10,10), was placed on the logit of g_0_, whereas an informative prior was used for the home-range-scale parameter σ. To incorporate this, we used an informative prior of gamma (40,35) for jungle cat and gamma (50,60) for rusty-spotted cat respectively. We also compared the density estimates using the informative priors with estimates of uninformative priors (e.g. U (0,50) prior for sigma) to understand the role of priors. We used 50,000 MCMC iterations (with the initial burning of 5,000) and the thinning rate of the chains was fixed as 1. As we estimated the parameters following iterations in the Bayesian framework, it was necessary to check the convergence of chains. Geweke diagnostic scores [30] were used to test the convergence of the MCMC chains in the “coda” package [31] in R 3.4 [32]. Geweke score of less than 1.6 indicated convergence of the estimated parameters in the chain.

### Habitat use

We investigated the habitat associations of the small cats using generalized linear models (GLM). We enumerated total counts at each site considering a 24-hour sampling interval as one sampling occasion and used this as a response variable. Data were pooled from all the four blocks for the analysis. Values of remotely sensed habitat variables; land use/landcover, evapotranspiration, forest-cover, elevation, normalised difference vegetation index (NDVI), distance from village, distance from waterbody were extracted from camera-trap locations with a 100-meter buffer and used as covariates for modelling the species count across sites. We restricted the buffer to 100 meters as we were interested in understanding the habitat preferences of species at a fine scale. Land use/landcover and forest cover were categorical variables while the remaining covariates were continuous variables (S1 Table). Forest cover was classified into very dense (cover >70%), moderately dense (cover 40–70%), open forest (cover 10–40%), scrub (cover <10%) and non-forest (www.fsi.nic.in). Similarly, land use/landcover was classified as human-habitation, agriculture, open forest, deciduous forest and waterbody (bhuvan.nrsc.gov.in). The main purpose of using these habitat variables was to model the species occurrence with habitat heterogeneity. Moreover, these variables were found to be effective predictors of small carnivore distribution from previous studies in India [18]. Evapotranspiration was used as a surrogate for aridity while forest cover and NDVI represented the canopy and vegetation cover of the habitat. Distance from village was used as a surrogate for human use.

We standardized all covariates using z-transformation and tested them for correlation. Significantly correlated variables (|r| <0.4) were dropped for further analysis. Generalized linear models were fitted with a “poisson” link function and model fitting was carried out in R 3.4 [32]. The best-fit model was chosen based on the difference between AIC of two models, (ΔAIC >2). We used model averaging for predictor variable coefficient estimate when multiple models were satisfying the ΔAIC criterion. Model averaging was carried out in “MuMIn” package in R 3.4 [32].

## Results

The total survey effort comprised of 10332 trap nights from 397 camera-traps. We photographed 23 mammals during the camera-trap survey including 171 photo-captures of jungle cat and 66 photo-captures of rusty-spotted cat. Of the 397 camera-trap locations, jungle cat was detected at 91 locations and rusty-spotted cat was detected at 38 locations respectively. Both the species were detected together at 9 locations.

**Fig 2:**
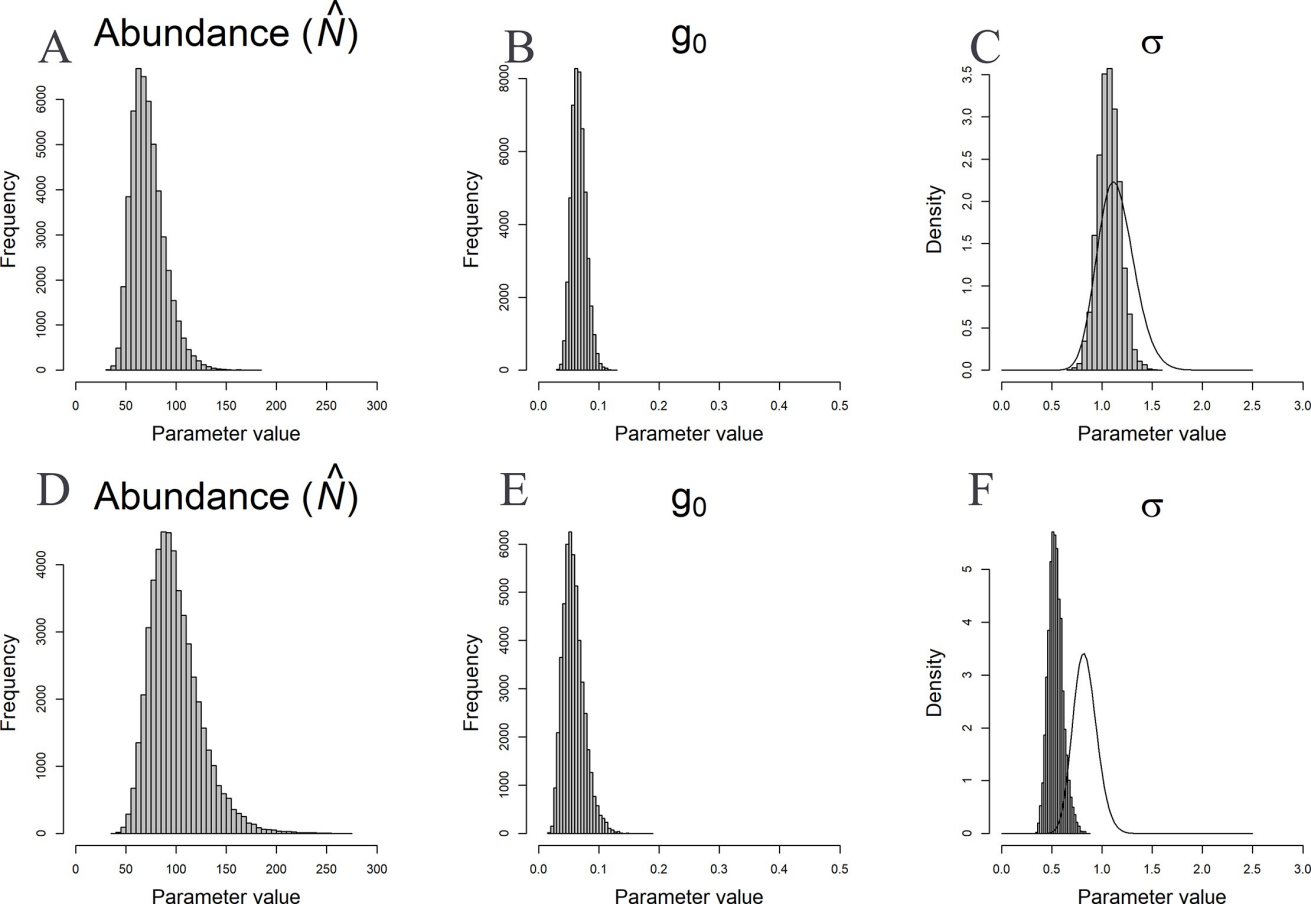
Posterior distributions of Jungle cat abundance (A) and Rusty-spotted cat abundance (D) (*N*^^^), and parameters of detection function (detection probability g_0_ (B & E), spatial-scale parameter σ (C & F)) using the spatial presence-absence model applied to jungle cat and rusty-spotted cat detections in camera traps from the Tadoba-Andhari Tiger Reserve. The solid line overlaid on the posterior distribution of σ is the prior gamma distribution used for the species. These estimates were derived following a Gamma (50,60) and (40,35) prior on σ for Rusty-spotted cat and Jungle cat respectively and uniform (-10,10) prior on g_0_.

The model estimated densities of 4.01 (95% CI 2.65–6.12) individuals/100 km^2^ for jungle cat and 6.67 (95% CI 4.07–10.74) individuals/100 km^2^ for rusty-spotted cat (Table 1). Extrapolating estimated density for the survey area, we estimated 72 (95% CI 48–111) jungle cat and 100 (95% CI 61–161) rusty-spotted cats in the tiger reserve. The baseline encounter rate (g_0_) and home-range scale parameter were greater for jungle cat than rusty-spotted cat (Table 1, Fig 2). There was no significant difference in density estimates with increase in buffer sizes (σ, 2σ and 5σ) for both the species (t(rusty) = 25.03, p<0.001 and t(jungle) = 7.84, p<0.001). Population estimates using uninformative priors overlapped with the informative priors for both the species but the coefficient of variation (CV) was much smaller with informative prior in case of Jungle cat. Informative priors had a CV of 22.4% whereas uninformative priors had a CV of 70.5% and for rusty-spotted cat CV was 25.8% for informative prior and 25.3% for uninformative prior. The density estimate for rusty-spotted cat was higher with the uninformative prior while the density estimate of jungle cat was similar.

**Table 1:** Parameter estimates of population size N, density and parameters of detection function (detection probability g_0_, spatial-scale parameter σ) from the spatial presence/absence model are given in the table.

	No. of independent captures	Density per 100 sq.km (95% CI)	Posterior N (95% CI)	Sigma estimate (95% CI)	g_0_ estimate (95% CI)
Jungle cat	171	4.01 (2.65–6.12)	72 (48–111)	1.069(0.858–1.294)	0.067 (0.046–0.092)
Rusty-spotted cat	66	6.67 (4.07–10.74)	100 (61–161)	0.539(0.412–0.703)	0.058 (0.030–0.099)

The Geweke diagnostic scores reflected convergence of all parameters of SPA models for both the species as the z statistic values was <1.6. Also, we tested the geweke scores of each parameter against z = 1.6 and reported the p-value, which was found to be significantly different for all the parameters. The geweke scores with the p values in the parenthesis of the jungle cat SPA models are given by, sigma = 1.17 (0.86), g_0_ = -0.94 (0.824), psi = -1.198 (0.09), N = -1.26 (0.073) while the geweke scores of the rusty-spotted cat SPA models are given by, sigma = -1.02 (0.16), g_0_ = 1.55 (0.94), psi = -0.77 (0.22), N = -0.72 (0.24).

**Fig 3:**
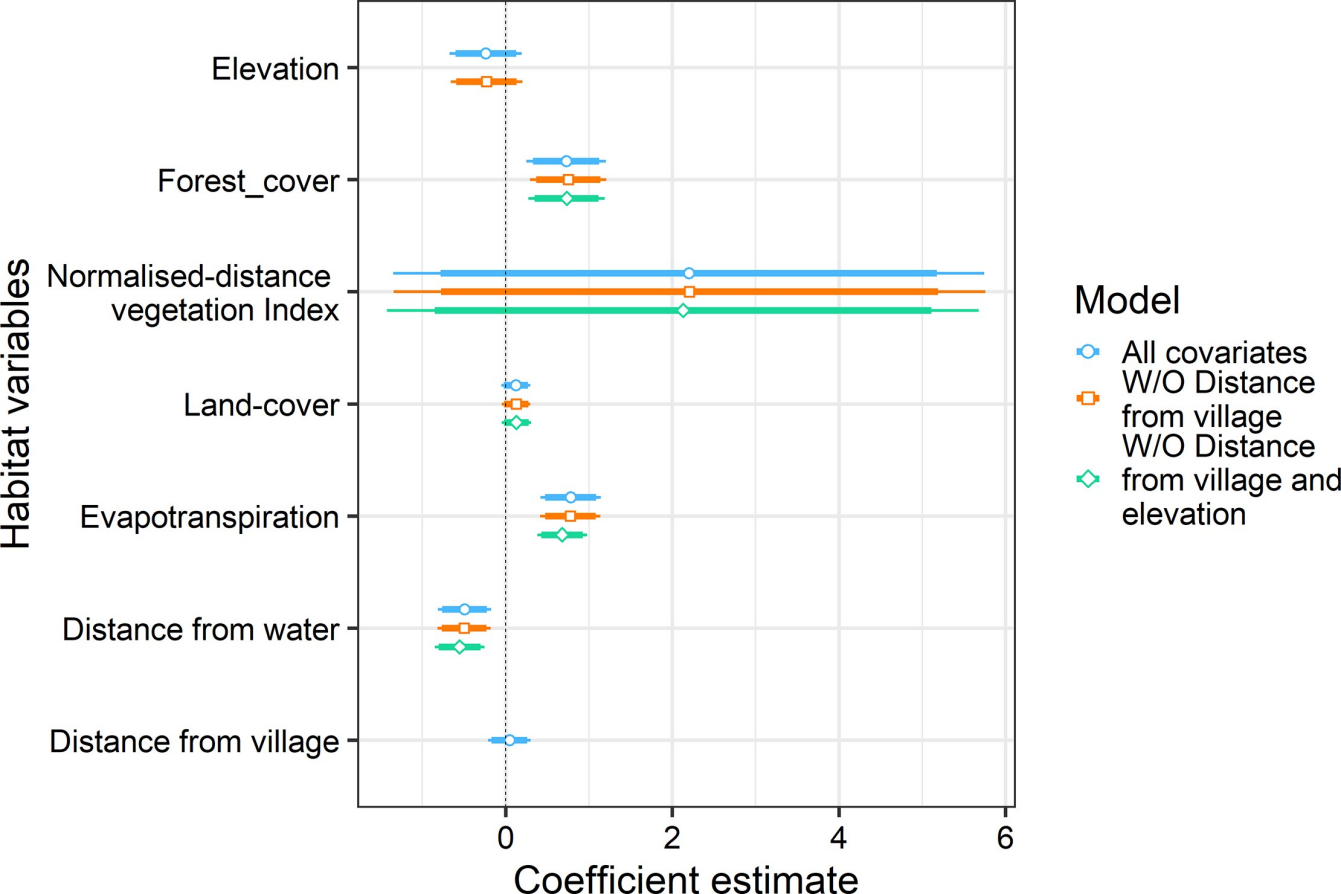
Scaled coefficients with standard deviation of habitat covariates from generalized linear model estimates for rusty-spotted cat. Broad horizontal lines depict the 95% confidence interval of each variable, while the narrow lines represent 90% confidence interval. Non-significant habitat variables, dropped from subsequent equations are represented by absence of the coefficient and standard deviation line.

**Fig 4:**
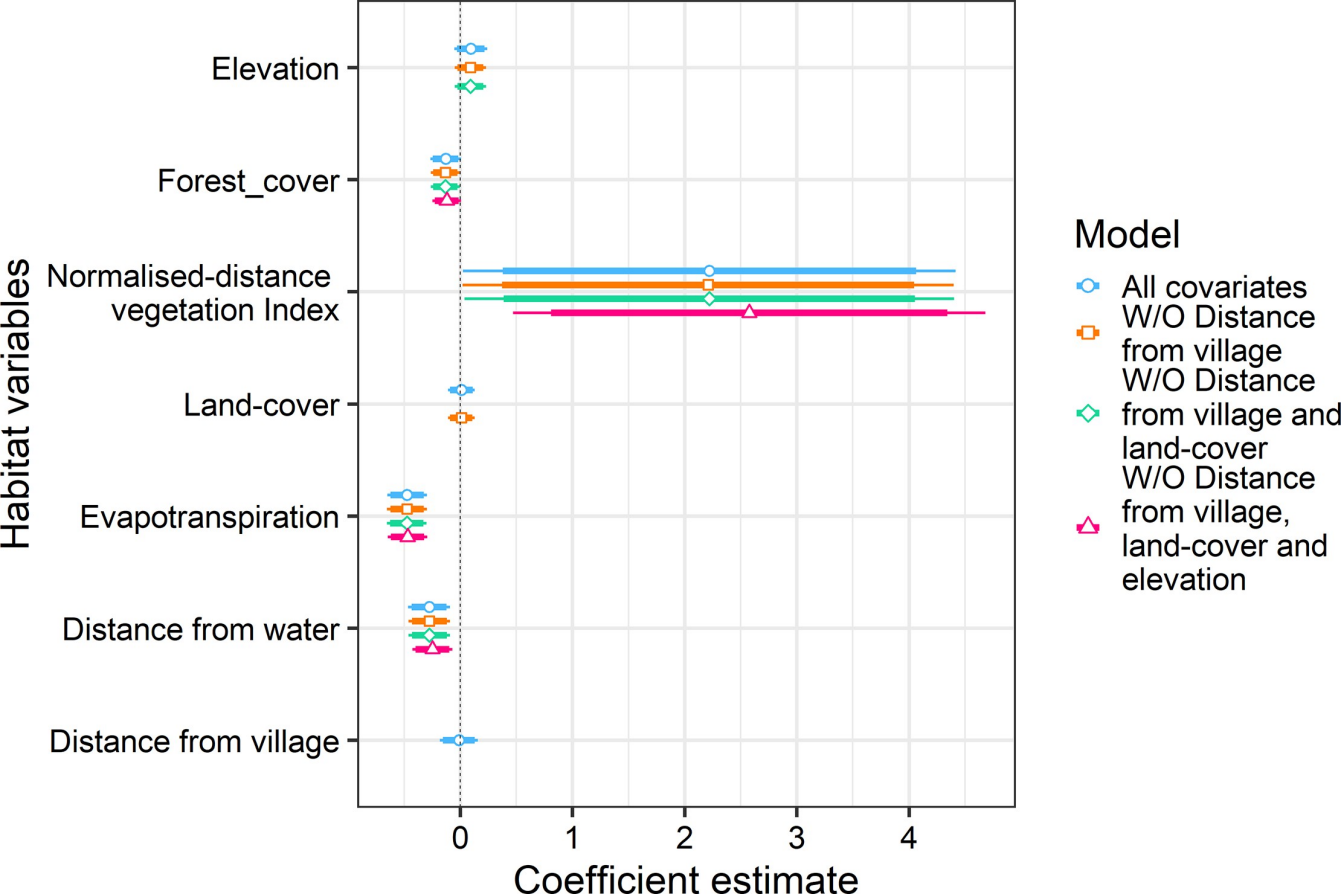
Scaled coefficients with standard deviation of habitat covariates from generalized linear model estimates for jungle cat. Broad horizontal lines depict 95% confidence intervals of each variable, while the narrow lines represent 90%. Non-significant habitat variables, dropped from subsequent equations are represented by the absence of the coefficient and standard deviation line.

Habitat use patterns using the generalized linear modelling reflected differential habitat use by both species. Coefficients of the predictor variables were generated using model-averaged equations satisfying the criteria ΔAIC <2. The occurrence of rusty-spotted cat was best predicted by forest cover (0.72 ± (SE) 0.24), evapotranspiration (0.71 ± (SE) 0.18) and distance from water (-0.53 ± (SE) 0.16) (Table 2, Table 3, Fig 3). Jungle cat occurrence was associated with NDVI (2.33 ± (SE) 1.12), evapotranspiration (-0.47 ± (SE) 0.09) and distance to water (0.27 ± (SE) 0.09) (Table 2, Table 4, Fig 4). The coefficient of the significant variables reflected the varied habitat preference for the species. Jungle cat preferred xeric habitats with negative association with forest cover while rusty-spotted cat was positively associated with dense forest cover and more humid areas.

**Table 2:** GLM coefficient with associated standard error values (in parenthesis) depicting habitat associations of the jungle cat and rusty-spotted cat. The coefficients and the associated standard errors were derived by averaging suitable models satisfying the ΔAIC <2 criteria.

Model covariate	Jungle cat	Rusty-spotted cat
Forest cover	-0.13 (0.067) ^+^	0.73 (0.24) **
Normalised difference vegetation index	2.34 (1.12) *	2.17 (1.80)
Evapotranspiration	-0.47 (0.09) ***	0.71 (0.18) ***
Distance from water	-0.27 (0.09) **	-0.53 (0.16) **
Landuse/ landcover	0.002 (0.034)	0.13 (0.09)
Distance from villages	-0.002 (0.047)	0.03 (0.13)
Elevation	0.06 (0.07)	-0.23 (0.22)

(Significance codes

*** < 0.001

** < 0.01

* < 0.05

^+^ < 0.1)

**Table 3:** Result of generalized linear models used to evaluate the habitat use patterns of rusty-potted cat based on remotely sensed habitat covariates around camera trap locations. elev- elevation; aet- actual evapotranspiration; lulc—Landuse/landcover, fcm- forest cover; ndvi–Normalised Difference Vegetation Index; waterdist- distance from water; villdist–distance from villages.

Covariates	Degrees of freedom	AICc	ΔAIC	weight
fcm+lulc+ndvi+aet+waterdist	6	395.34	0.00	0.25
fcm+lulc+elev+aet+waterdist	6	395.63	0.29	0.22
fcm+lulc+ndvi+elev+aet+waterdist	7	396.21	0.87	0.16
fcm+ndvi+elev+aet+waterdist	6	396.57	1.23	0.14
fcm+lulc+villdist+aet+waterdist	6	396.68	1.34	0.13
fcm+lulc+ndvi+villdist+aet+waterdist	7	397.57	2.03	0.09

**Table 4:** Result of generalized linear models used to evaluate the habitat use pattern of jungle cat based on remotely sensed habitat covariates around camera trap locations. elev- elevation; aet- actual evapotranspiration; lulc-Landuse/landcover, fcm- forest cover; ndvi–Normalised Difference Vegetation Index; waterdist- distance from water; villdist–distance from villages.

Covariates	Degrees of freedom	AICc	ΔAIC	weight
fcm+elev+ndvi+aet+waterdist	6	395.34	0.00	0.38
fcm+villdist+ndvi+aet+waterdist	6	395.63	1.51	0.18
fcm+lulc+ndvi+aet+waterdist	6	396.21	1.55	0.17
fcm+lulc+ndvi+elev+aet+waterdist	7	396.57	2.04	0.14
fcm+villdist+ndvi+elev+aet+waterdist	7	396.68	2.06	0.13

## Discussion

This study attempted to estimate the population of two sympatric and individually unidentifiable small cat species and understand their habitat preference. We report the first ever population density estimates of jungle cat and rusty-spotted cat that ranges from 3–7 individuals per 100 km^2^. The estimates, based on the largest camera trapping dataset available for these small cat species, are comparable to density estimates of other small cats [33–36]. The estimated densities of these elusive cats can significantly contribute to conservation strategies. We also observed fine-scale spatial segregation supporting our hypothesis, facilitating co-occurrence between these small cat species.

### Population density estimates

There are no previous records of population density estimates for both of the studied species. But estimates of this study are comparable to population density of other individually identifiable small cats of comparable body sizes, the leopard cat (*Prionailurus bengalnensis*) [33, 34] and the marbled cat (*Pardofelis marmorata*) [35,36]. Contrary to the non-spatial models, spatial models provide site-specific detection probability and a scale of animal space use (sigma). Estimates of the movement parameter (sigma) of this study are comparable with that of leopard cat [34] and marbled cat [35] but smaller than the sigma estimates of leopard cat [33] or marbled cat [36]. Difference in the analytical procedure (spatial presence-absence vs spatial capture-recapture) prevented direct comparison of the parameter estimates. In our study, the number of captures of jungle cat was greater but their density estimate was lower than that of rusty-spotted cat. This was attributed to the assumption of a larger home-range of jungle cat compared to rusty-spotted cat. Earlier studies found the density of small cats reflected top-down competition from other large predators present as has been evidenced in a study on leopard cats from the Western Ghats in India [34]. Moreover, when the carnivore community remained similar, species density was found to be associated with other biotic factors. Studies on neotropical small cats (ocelot) revealed dependence on primary productivity [37], density estimates ranging from 2.3 per 100 km^2^ (Chiquibul, Belize) to 94.7 per 100 km^2^ (Peruvian Amazon) [38, 39]. Future studies should consider these factors along with the presence of other carnivores as covariates to model density of these small cat species.

### Habitat use patterns

We found evidence supporting our hypothesis that there exists fine-scale habitat segregation between the two sympatric cat species. Although the species have a majorly overlapping distribution range in the Indian subcontinent, at a fine-scale of camera trap sampling sites they hardly cooccur. The factors that significantly affected rusty-spotted cat occurrence were those associated with dense forest while jungle cat occurrence was associated with open forests and scrublands. Our results reported a similar pattern as reported by Kalle et al. [18] from the Western Ghats where the presence of jungle cat and rusty-spotted cat was positively related to open forest and deciduous forest respectively. The affinity of rusty-spotted cat for deciduous forest supports the semi-arboreal adaptation of this species [18] which likely facilitates avoiding competition with other carnivores within similar habitats. Similar studies on sympatric meso-predators from other landscapes [40] found negligible spatial avoidance between species but adaptations of segregation in their activity patterns. The fine-scale segregation in habitat niches might have aided in the evolution of sympatry between these species with overlapping distribution ranges.

### Caveats and limitations

Although the population estimation and habitat use pattern in our study where based on a robust analytical framework, there are certain inevitable limitations similar to other field studies. We could not employ a finer grid size for camera-trap sampling due to logistical constraints. Prior distributions for the spatial count model were selected based on the data available for leopard cat [23] and allometric equations of daily-movement, home-range and body mass. Radio-telemetry studies can aid in providing refined estimates for the prior distributions and robust estimates using spatial capture-recapture [7] or spatial mark-resight [41] framework. Further studies can evaluate the efficacy of the spatial presence-absence model by comparing estimates from other frameworks. Alongside remotely sensed environmental variables, micro-habitat variables collected from sampling sites can be collated to understand multi-scale habitat preferences. The presence of other species as a predictor variable can also reveal the effect of species interaction in shaping habitat use patterns. The study was restricted to a tiger reserve whereas both the study species are also known to occur outside protected areas. It would be useful for future studies to look at density estimates across various gradients of natural and anthropogenic disturbances and varied mammal community as well.

## Conclusions

Lack of proper baseline data and ordinated conservation policies have led to local extinction of species from varied habitats globally [42, 43]. Reliable estimates of species abundance and knowledge of their habitat preference are of fundamental importance as they facilitate management goals and policy decisions for successful conservation of species. The understanding of the unique ecological role of small carnivores [44] can be adequately supplemented by baseline data as provided by our study. In addition to providing the estimates of the population density of two sympatric small cats that cannot be individually identified, our study demonstrates the utility of spatial count models for the population estimation of unidentifiable species. The study highlights the importance of different habitat types explaining fine-scale habitat segregation between co-occurring species. With carefully designed field surveys and maintaining proper caution in the generalisation of results, our model may be extended and applied to other species which lack individually identifiable morphological features like pelage patterns (such as civets, bears, and foxes) and are cryptic and elusive besides being of high conservation priority. Trends of population estimates and habitat use from this study can contribute to the assessment of conservation status and devising mitigation principles. Long term studies assessing other life-history parameters of small cats are imperative in our understanding of their ecological role in carnivore communities thriving across varied landscapes.

## Supporting information

S1 Table Details of the remotely sensed variables used as covariate in the occupancy framework to evaluate the habitat use of small cats in Tadoba-Andhari Tiger Reserve. (DOCX)
