# Supplementary material for: Population density and habitat use of two sympatric small cats in a central Indian reserve
Source: PLoS One. 2020 Jun 4;15(6):e0233569. doi: 10.1371/journal.pone.0233569 (PMC7271992; doi:10.1371/journal.pone.0233569)
Supplement: S1 Table — (DOCX) [file pone.0233569.s001.docx]

S1 Table. Details of the remotely sensed variables used as covariate in the occupancy framework to evaluate the habitat use of small cats in Tadoba-Andhari Tiger Reserve

| Habitat Covariate | Type | Source |
| --- | --- | --- |
| Elevation | Continuous | SRTM (http://srtm.csi.cgiar.org) |
| Forest Cover | Categorical | Forest Survey of India (http://fsi.nic.in) |
| Landuse-landcover | Categorical | Bhuvan (http://bhuvan.nrsc.gov.in) |
| Actual evapotranspiration | Continuous | CGIAR-CSI (https://cgiarcsi.community/) |
| Normalized Difference Vegetation Index | Continuous | Sentinel-2 (https://scihub.copernicus.eu/)* |
| Distance from villages | Continuous | Maharashtra Remote Application Center (MRSAC) http://www.mrsac.gov.in/en/home |
| Distance from waterbody | Continuous | DIVA GIS (http://www.diva-gis.org) |

* Calculated using the formula with the respective bands, ndvi = (near infra-red - red)/ (near infra-red + red)
